# Role of Weak Materials in Earthquake Rupture Dynamics

**DOI:** 10.1038/s41598-019-43118-5

**Published:** 2019-04-29

**Authors:** Tetsuro Hirono, Kenichi Tsuda, Shunya Kaneki

**Affiliations:** 1 Department of Earth and Space Science, Graduate School of Science, Osaka University, Toyonaka, Osaka 560-0043 Japan; 2 Center for Safety and Reliability Engineering, Institute of Technology, Shimizu Corporation, Koto, Tokyo, 135-8530 Japan; 3 Present Address: Disaster Prevention Research Institute, Kyoto University, Uji, Kyoto 611-0011 Japan

**Keywords:** Structural geology, Seismology

## Abstract

Weak materials in seismic slip zones are important in studies of earthquake mechanics. For instance, the exceptionally large slip during the 2011 Tohoku-Oki earthquake has been attributed to the presence of smectite in the fault zone. However, weak fault rocks cannot accumulate large amounts of elastic strain, which is thought to counter their ability to enhance seismic rupture. It is well known that if the permeability of a weak fault is low enough to allow friction-induced thermal pressurization of interstitial fluid, the fault strength decreases dramatically. However, whether intrinsic weakness of fault material or thermal pressurization more efficiently produces large slip on faults bearing weak materials has not been determined. To investigate the role of weak materials in earthquake rupture dynamics, we conducted friction experiments and dynamic rupture simulations using pure smectite and pure graphite to represent weak fault materials. Even when we assumed no thermal pressurization, simulated faults in both media were able to trigger large slip because their extremely low friction was insufficient to arrest the inertial motion of rupture propagating along the fault. We used similar rupture simulations to investigate the cause of the huge slip near the trench during the 2011 Tohoku-Oki earthquake and demonstrated that it can be attributed to thermal pressurization, although our findings suggest that the presence of smectite in the plate-boundary fault may also be required.

## Introduction

Our knowledge of the frictional strength of faults, which is the predominant control of rupture dynamics and earthquake magnitude, has benefited from almost half a century of laboratory experiments on rocks^1,2^. Because weak materials such as phyllosilicates and graphite have low frictional resistance^3,4^, their presence is thought to weaken faults in the brittle regime^5–7^ and to account for fault creep such as that observed along the San Andreas Fault in California^6,7^. The exceptionally large fault slip near the axis of the Japan Trench (50–80 m) during the 2011 Tohoku-Oki earthquake^8,9^ has been attributed to the presence of low-friction smectite^10–12^. However, there is evidence that clay minerals cannot accumulate sufficient elastic strain/stress to cause such a large fault slip^13^ and that their presence also stabilizes the slip^3,14^. The lack of seismicity and fault rupture along the shallow parts of plate subduction boundaries (shallower than 5–15 km) has been attributed to the presence there of subducting clayey sediments^15,16^. However, recent friction experiments simulating subduction driving rates of centimetres per year revealed unstable slip behaviour in clayey faults that would favour earthquake rupture^17^. Despite numerous studies during recent decades on the effect of weak materials on earthquake mechanisms, it is still not well understood.

**Figure 1:**
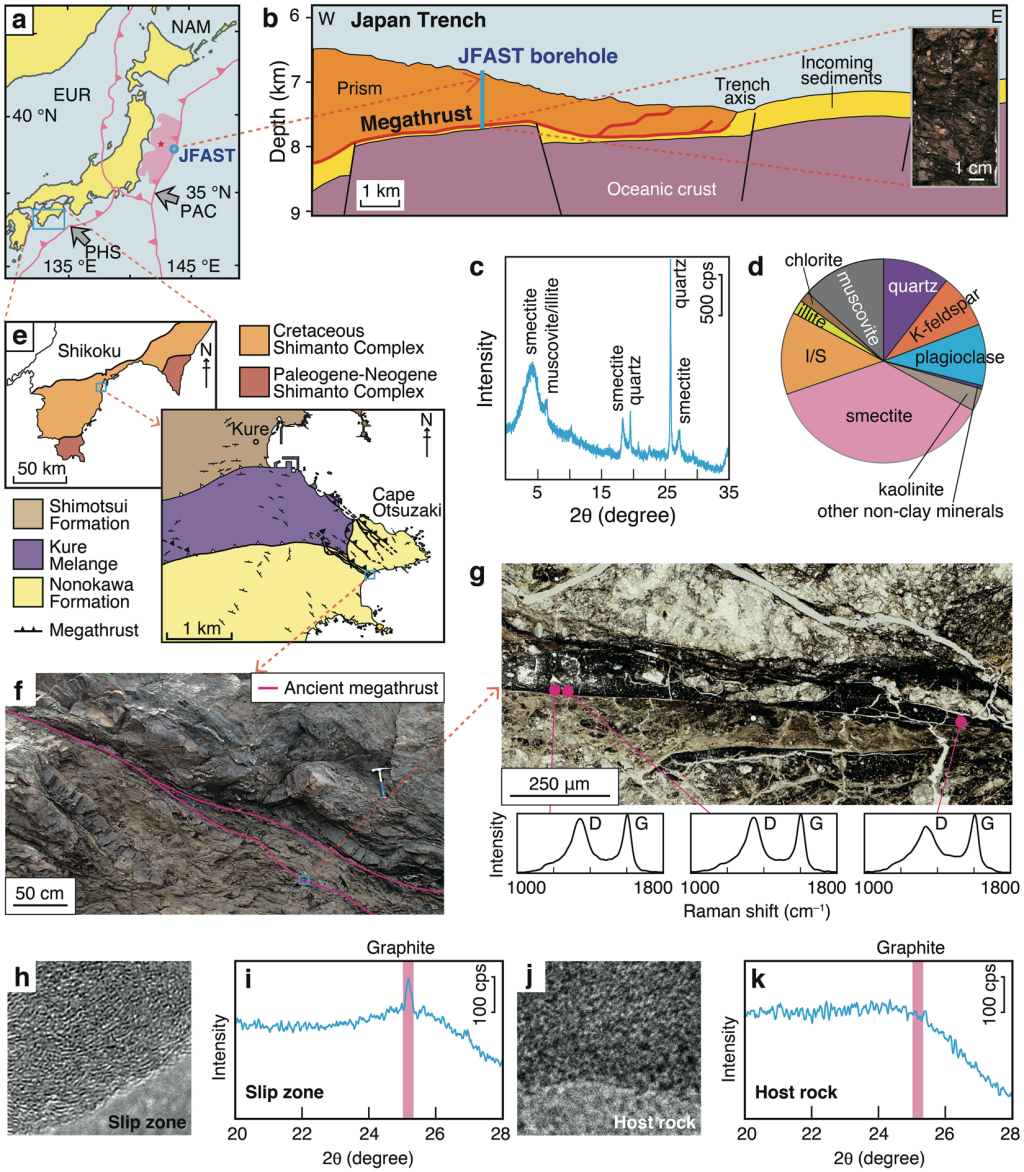
Natural fault zones containing weak materials in the Japan Trench and Shimanto accretionary complex. (**a**) Tectonic setting and location of Japan Trench Fast Drilling Project (JFAST)^10^. Pink shading, rupture area of the 2011 Tohoku-Oki earthquake; red star, 2011 hypocentre; open blue circle, JFAST borehole site. Pink lines show major tectonic plate boundaries: NAM, North America plate; PAC, Pacific plate; EUR, Eurasia plate; PHS, Philippine Sea plate. (**b**) Cross section across the Japan Trench showing the location of the JFAST borehole site. Photograph shows plate-boundary fault material retrieved from the borehole. (**c**,**d**) Results of XRD analysis of the fault material. (**e**) Maps showing the location of an ancient subduction megathrust in the Shimanto accretionary complex^20^, and (**f**) field photograph of the megathrust. (**g**) Microscopic image of the ancient megathrust and Raman spectra showing disordered bands (D, 1360 cm^−1^) and graphite bands (G, 1600 cm^−1^). (**h**,**i**) TEM image and XRD pattern of carbonaceous material from the slip zone. (**j**,**k**) TEM image and XRD pattern of carbonaceous material from intact host rock.

In this study, we investigated the role of weak materials (represented by smectite and graphite) in earthquake rupture dynamics. To investigate a smectite-bearing slip zone we numerically modelled a plate-boundary thrust in the Japan Trench (ca. 40–80 wt.% bulk smectite content^11,18^) (Fig. 1a–d). Although graphite-bearing slip zones have been observed previously only in inland faults (e.g., the Longmenshan fault, China^19^), we discovered a graphite-bearing slip layer within a fossil subduction-boundary megathrust in the Shimanto accretionary complex, Japan (Fig. 1e,f). The slip layer there is about 100 µm thick^20^ and is composed of carbonaceous material, as indicated by the presence of graphitic and disordered bands in Raman spectra (Fig. 1g). The carbonaceous material was confirmed to be graphite by the presence of clear graphite lattice fringes under transmission electron microscopy (TEM) and a sharp X-ray diffraction (XRD) peak representing the graphite (002) plane at 25.1° 2θ (3.548 Å) (Fig. 1h,i). In contrast, the surrounding host rocks showed a maze-like TEM pattern and no graphite XRD peak (Fig. 1j,k). Thus, both graphite-bearing and smectite-bearing slip zones may play important roles during both subduction and inland earthquakes.

## Results and Discussion

### Friction properties of weak materials

**Figure 2:**
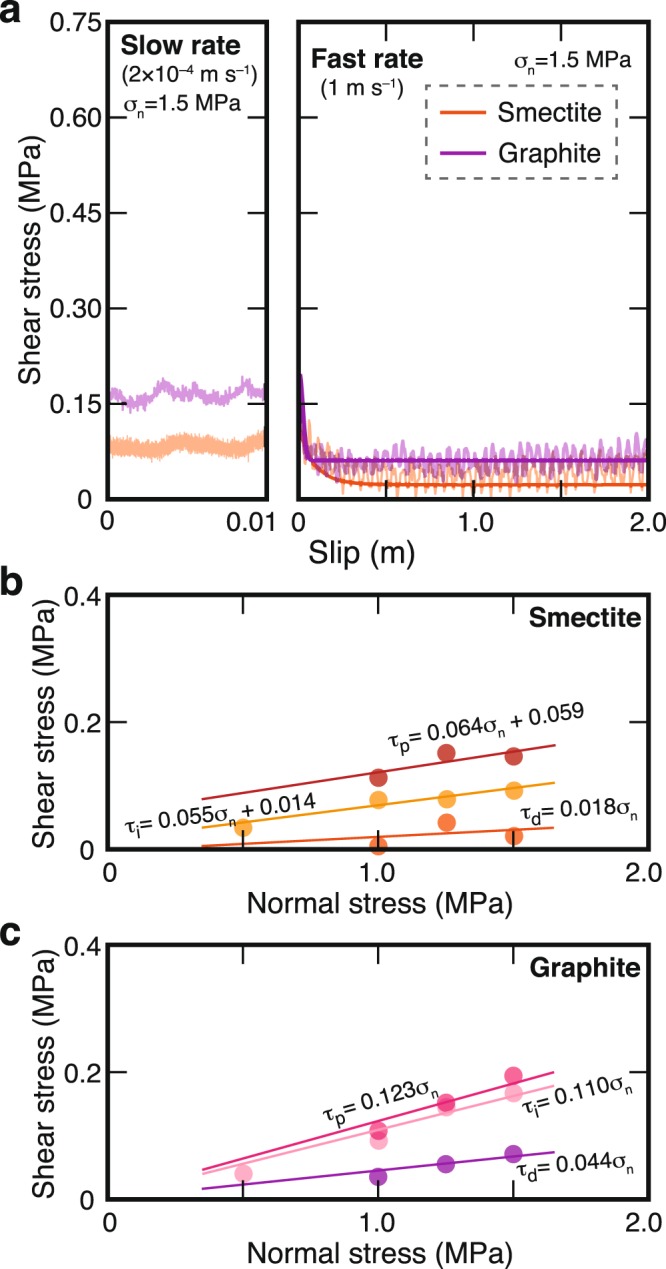
Determination of frictional properties of smectite and graphite. (**a**) Evolution of shear stresses of synthetic smectite- and graphite-bearing slip zones as functions of displacement (slip) under normal stress (*σ*_n_) of 1.5 MPa at slow slip rate (left) and high slip rate (right). (**b**,**c**) Plots of initial shear strength (*τ*_i_), peak shear stress (*τ*_p_), and dynamic shear stress (*τ*_d_) versus *σ*_n_ for slip zones composed entirely of (**b**) smectite and (**c**) graphite.

To experimentally evaluate the effects of smectite and graphite on seismic rupture processes, we performed rotary-shear friction experiments to quantify their frictional properties (see Methods). The resultant shear stress (*τ*) was plotted versus slip (*D*) under 1.5 MPa normal stress (*σ*_n_) (Fig. 2a). We determined the friction coefficients at the slow slip rate (*µ*_i_) by averaging the values of *τ*/*σ*_n_, and those at the fast slip rate by power-law fitting according to the relationship *τ* = (*µ*_d_ − (*µ*_d_ − *µ*_p_)exp(−*D*/*D*_c_))*σ*_n_, where *µ*_d_ and *µ*_p_ are dynamic and peak friction coefficients, respectively, and *D*_c_ is critical slip distance (Supplementary Fig. S1). The initial stress state (representing the stress state just before earthquake rupture) was unknown, but a previous numerical study determined it from the friction coefficient at slow slip rates^18^.

For both the smectite and graphite samples, the dependence of shear stress (*τ*) on normal stress (*σ*_n_) showed high linearity. We determined the friction coefficients, *µ*_i_, *µ*_p_, and *µ*_d_ of smectite to be 0.055, 0.064, and 0.018, respectively (Fig. 2b), and those of graphite to be 0.110, 0.123, and 0.004, respectively (Fig. 2c).

### Simulations of dynamic rupture propagation

**Figure 3:**
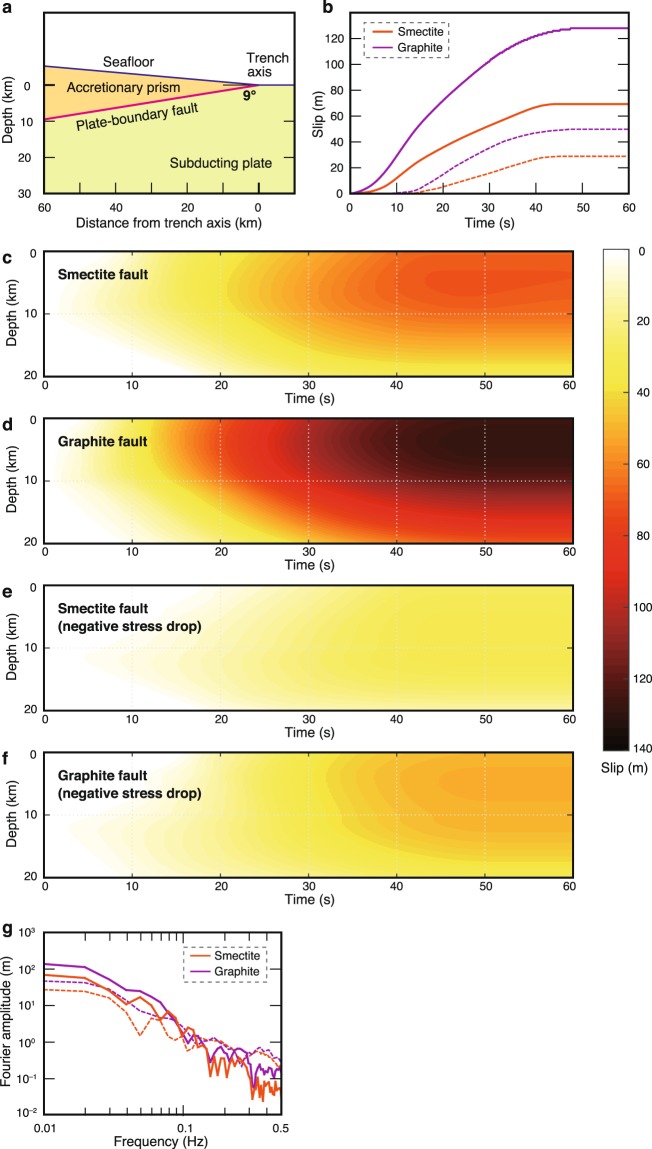
Spatiotemporal distribution of near-trench earthquake slip for weak faults. (**a**) Fault geometry used in dynamic rupture modelling. (**b**) Accumulation of slip with time at 2 km depth on the fault. (**c**–**f)** Spatiotemporal evolution of slip for fault rocks composed entirely of smectite and graphite without thermal pressurization: (**c**,**d**) with a positive stress drop determined by our friction experiments and (**e**,**f**) with a negative stress drop of −0.2 MPa. (**g**) Fourier amplitudes of resultant slip rates at 2 km depth on the faults. In (**b**,**g**) solid lines indicate modelling with a positive stress drop; dashed lines indicate modelling with a negative stress drop (−0.2 MPa).

To understand how low friction of weak materials affects the process of seismic rupture and the amount of slip, we simulated two-dimensional dynamic rupture propagation on the basis of a slip-weakening friction power law^21^. Numerical models coupled with the rate-and-state friction law^22^ have been used to reproduce seismic cycles for subduction environments^23–25^. However, this approach comes at the expense of failing to account for experimental data from actual fault rocks. Therefore, we ran simulations for fault rocks composed entirely of either smectite or graphite by using our experimentally obtained friction parameters. By applying the spectral-element method^21^ (see Methods) to a realistic fault geometry of the Japan Trench (Fig. 3a), a uniform elastic medium with a grid spacing of 1 km was assumed with a density of 2600 kg m^−3^, *P*-wave velocity of 6.30 km s^−1^, and *S*-wave velocity of 3.54 km s^−1^. For shear strength in the shallow part of the fault (1–10 km depth) we used the values we obtained in our friction experiments on smectite and graphite. An earthquake nucleation procedure was manually invoked at 11 km depth (see Methods) and the results of the computation showed the direction of rupture propagation and the evolution of slip along the fault. The dynamical model parameters are summarized in Supplementary Fig. S2.

Simulations for both the pure smectite and pure graphite fault rocks showed large slip distances near the trench (about 70 and 130 m at 2 km depth on the faults, respectively; Fig. 3b–d), even though simulated without thermal pressurization. Furthermore, even if their values of *µ*_i_ were assumed to be lower than those of *µ*_d_ (i.e., simulation with −0.2 MPa negative stress drop that characterizes rupture dynamics and radiated seismic energy, Supplementary Fig. S1), they also showed large slip distances of about 30 and 50 m for the smectite and graphite fault rocks, respectively (Fig. 3b,e,f). Although absolute values of normal and shear stresses might affect the magnitude of the resultant slip distance, additional simulations for which we assumed a rock density of 2900 kg m^−3^ at 1 km depth and the same magnitudes of stress drop to those used at all depths in the original simulations also showed large slip distances near the trench for the smectite and graphite faults (about 70 and 100 m at 2 km depth, respectively: Supplementary Fig. S3). The resulting frequency spectra for slip rate (which represents the spectra of seismic waves radiated from the fault) near the trench showed less-pronounced high frequency ranges (>0.1 Hz) (Fig. 3g).

On the other hand, friction coefficients previously determined for plate-boundary fault rocks in the Japan Trench are *µ*_p_ = 0.280 and *µ*_d_ = 0.170 (ref.^11^), which are higher than those for our pure smectite, probably because the fault rocks in the Japan Trench contain high-friction components such as quartz and feldspar (ca. 20 wt.%)^11^. However, if thermal pressurization occurred during frictional slip, fault strength would be dramatically weakened^26^. Coefficients of friction of *µ*_p_ = 0.170 and *µ*_d_ = 0.005 for fault rocks in the Japan Trench have been determined on the basis of thermal pressurization modelling and the *in situ* physical properties of the rocks^18^. Undrained friction experiments, in which thermal pressurization is likely to occur, have also shown ultralow friction: *µ*_p_ = 0.1 and *µ*_d_ = 0 (ref.^11^), *µ*_p_ = 0.12 and *µ*_d_ = 0.07 (ref.^27^). Thus, thermal pressurization can play an important role in fault slip, independent of the intrinsic frictional properties of the rocks. We therefore evaluated the evolution of slip near the Japan Trench with and without thermal pressurization.

For our simulations of rupture propagation near the trench during the 2011 Tohoku-Oki earthquake, we assumed that the rupture nucleated at 11 km depth on the plate-boundary fault and propagated updip. Although this depth is considerably shallower than the hypocentral depth (~24 km) estimated by the Japan Meteorological Agency for the 2011 Tohoku-Oki earthquake^28^, we focus here on the evolution of slip near the trench because the material properties of rocks at depths of around 24 km are unknown. By applying the spectral-element method^21^ to a realistic fault geometry of the Japan Trench (Fig. 3a) together with material data from previous reports on the plate boundary fault rocks^11,18,27,29^ (summarized in Supplementary Fig. S4), we computed the direction of rupture propagation and the evolution of slip along the fault.

**Figure 4:**
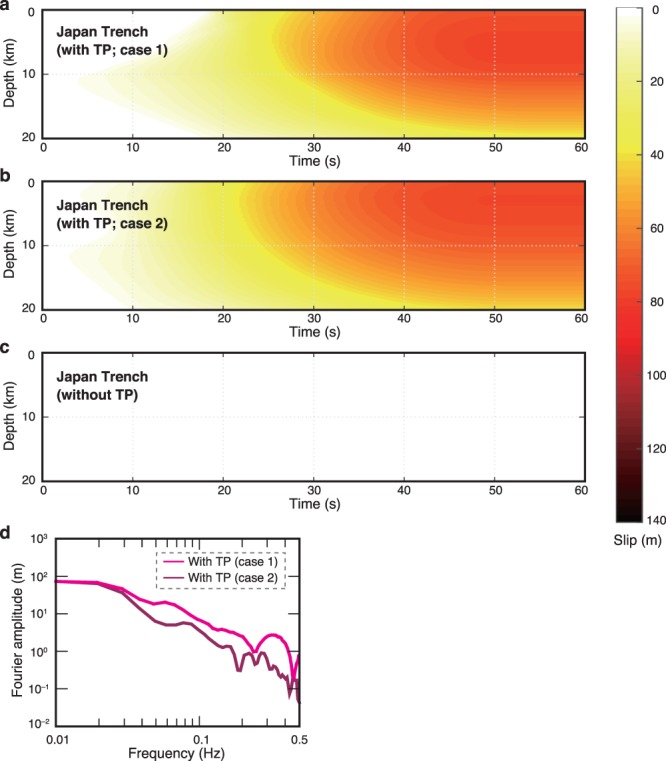
Spatiotemporal distribution of near-trench earthquake slip for Japan Trench. (**a**–**c**) Spatiotemporal evolution of slip on the fault taking account of thermal pressurization (TP) by using friction parameters from (**a**) ref.^18^ (case 1), (**b**) ref.^27^ (case 2), and (**c**) without TP (friction parameters from ref.^11^). (**d**) Fourier amplitudes of resultant slip rates at 2 km depth on the fault.

For the cases in which thermal pressurization occurred at the plate-boundary fault, large slips of about 75–77 m near the trench were obtained (Fig. 4a,b); this amount of slip near the trench is consistent with slips previously reported for the 2011 Tohoku-Oki earthquake (50–80 m slip^8,9^). However, the case without thermal pressurization showed almost zero slip along the fault (Fig. 4c), thus indicating the presence of a barrier to propagation of rupture updip from the deep part of the fault, likely a result of the negative stress drop because *µ*_d_ (0.170)^11^ was higher than *µ*_i_ (0.090)^29^. If the dynamic shear stress is higher than the initial shear stress, forward propagation of the rupture front becomes difficult^30^. Thus, we suggest that thermal pressurization (not the frictional properties of the fault rock) was the principal mechanism that produced the large slip near the trench during the 2011 Tohoku-Oki earthquake. The resulting frequency spectra for slip rate near the trench (Fig. 4d) show less-pronounced high frequency ranges (>0.1 Hz), which is also consistent with the observed radiation from the shallow area of large slip during the 2011 Tohoku-Oki earthquake^31^.

### Implications for rupture dynamics on weak faults

Fault creep has been attributed to the presence of weak materials in fault rocks, for instance, talc-bearing serpentinite or smectite in fault gouge in the San Andreas Fault^6,7^. Creeping sections have been reported to provide barriers to rupture propagation into the southern and northern locked parts of the San Andreas Fault^32^. However, our simulations showed that even though weak materials in faults cannot accumulate large amounts of elastic strain they can accommodate large seismic ruptures because of their extremely low shear strengths, which are insufficient to arrest the inertial motion of rupture propagating along a fault. A numerical simulation of long-term seismic activity (in which stable, velocity-strengthening behaviour at low slip rates was combined with coseismic weakening due to thermal pressurization) indicated that unstable large slips can occur in segments that usually creep^23^. However, our study provides the first clear evidence that, in addition to coseismic weakening, extremely low friction of fault materials can be a principal mechanism for producing exceptionally large fault slips. Differences in the amounts of weak materials in localized slip zones within a fault might determine whether fault slip is enhanced or reduced, because more weak material promotes lower friction (e.g., a slip zone composed entirely of smectite versus ca. 40–55 wt.% smectite^18,27^ in Japan Trench material).

The results of our integrated analyses of frictional properties of fault materials and dynamic rupture simulations will contribute to future quantitative evaluations of near-trench slip at plate boundary faults and may be applicable in future earthquake disaster mitigation studies in subduction zone settings. To further our understanding of the entirety of earthquake mechanics (i.e., earthquake nucleation and rupture propagation) in faults bearing weak materials, further research is needed on frictional stability as expressed by the rate-and-state friction law^22^ for a range of slip rates including ultralow subduction-driving slip rates^17^, and more comprehensive modelling is needed taking into consideration both nucleation physics and rupture propagation simulations similar to those presented here.

## Methods

### Rotary-shear friction experiments

A rotary shear apparatus was used for our friction experiments (Supplementary Fig. S5). We used pure smectite (SWy-s, Clay Mineral Society repository) and pure graphite (Wako Pure Chemical Industries). All sample materials were first sieved to isolate particles smaller than 75 µm. About 2 g of powdered wet sample was then placed between the ends of two Berea-sandstone cylinders and sealed in a polytetrafluoroethylene sleeve to prevent leaks. Because the sandstone is permeable, the interstitial fluid pressure in the sample was considered to be low^11^. Time series of shear stress profiles (slip) were recorded at slip rates of 2 × 10^−4^ (slow) and 1 m s^−1^ (fast) after correction for friction between the sandstone cylinders and the sleeve. Axial loads applied during rotary shearing were 0.5, 1.0, 1.25, or 1.5 MPa.

### Dynamic earthquake rupture modelling

We used the spectral element method (SEM2DPACK)^21^ on the basis of a slip-weakening law^33^ for our modelling of dynamic rupture propagation. The relation of this modelling to rate- and state-dependent friction (RSF), established by experimental rock friction results at relatively low slip rates^22^, has been thoughtfully described in previous research^18^. Although RSF has been applied to explain fault behaviour during shallow, slow earthquakes along plate interfaces^34,35^, dramatic weakening (e.g., due to thermal pressurization) can dominate RSF weakening during earthquake nucleation at slip speeds in excess of roughly 10^−4^ to 10^−2^ m s^−1^ (ref.^36^). During dynamic rupture, such high slip rates are rapidly exceeded soon after the passage of the rupture front, so RSF weakening is not dominant during the dynamic phase of seismic slip on crustal faults. Therefore, we applied a slip-weakening friction law in our modelling.

To manually nucleate rupture at 11 km depth, we used negative values of strength excess (i.e., peak stress – initial stress): −5 and −10 MPa for the smectite and graphite faults, respectively. The strength excess of −10 MPa we used at 11 km depth was the same as that used at that depth in a previous analysis of megaquakes in the Japan Trench^18^. All model parameters are summarized in Supplementary Figs S2 and S4.

## Supplementary information

Supplementary Information
