# Supplementary material for: Role of Weak Materials in Earthquake Rupture Dynamics
Source: Sci Rep. 2019 Apr 29;9:6604. doi: 10.1038/s41598-019-43118-5 (PMC6488621; doi:10.1038/s41598-019-43118-5)
Supplement: Supplementary file 1 — Supplementary Information [file 41598_2019_43118_MOESM1_ESM.pdf]

SUPPLEMENTARY INFORMATION

Role of weak materials in earthquake rupture dynamics

Tetsuro Hirono[1*], Kenichi Tsuda[2], and Shunya Kaneki[1,3]

[1] Department of Earth and Space Science, Graduate School of Science, Osaka University, Toyonaka, Osaka 560-0043, Japan.

[2] Center for Safety and Reliability Engineering, Institute of Technology Shimizu Corporation, Koto, Tokyo 135-8530, Japan.

[3] Present address: Disaster Prevention Research Institute, Kyoto University, Uji, Kyoto 611-0011, Japan.

* Corresponding author. Contact: hirono@ess.sci.osaka-u.ac.jp

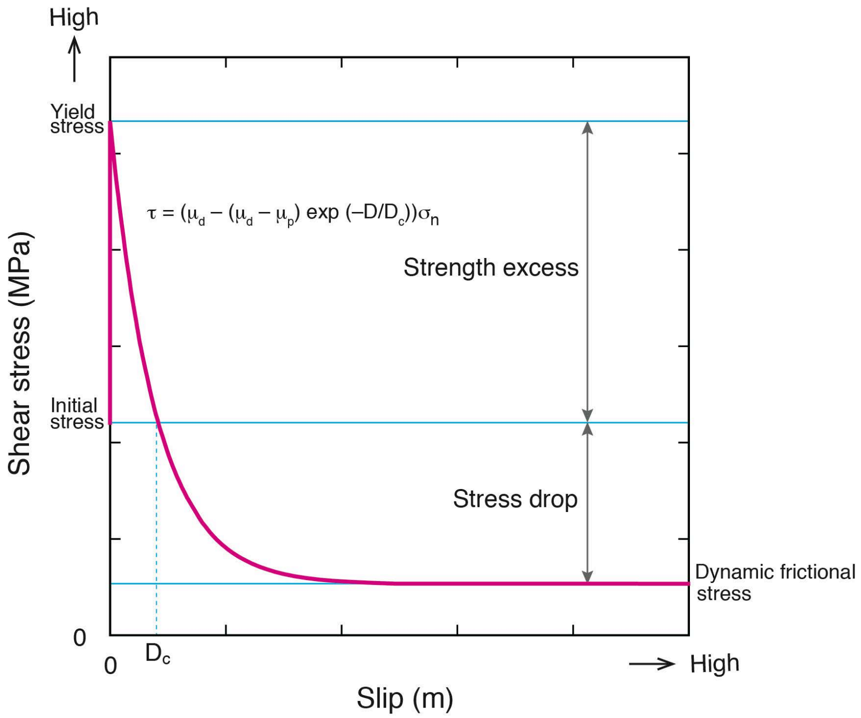

Supplementary Figure S1. Curve fitting for dynamic rupture modelling. Strength excess = yield shear stress – initial shear stress, and stress drop = initial shear stress – dynamic frictional stress. τ, shear stress; σ_n, normal stress, D, slip; μ_d, dynamic friction coefficient; μ_p, peak friction coefficient; D_c, critical slip distance. Positive and negative values of stress drop are referred to as "positive stress drop" and "negative stress drop", respectively.

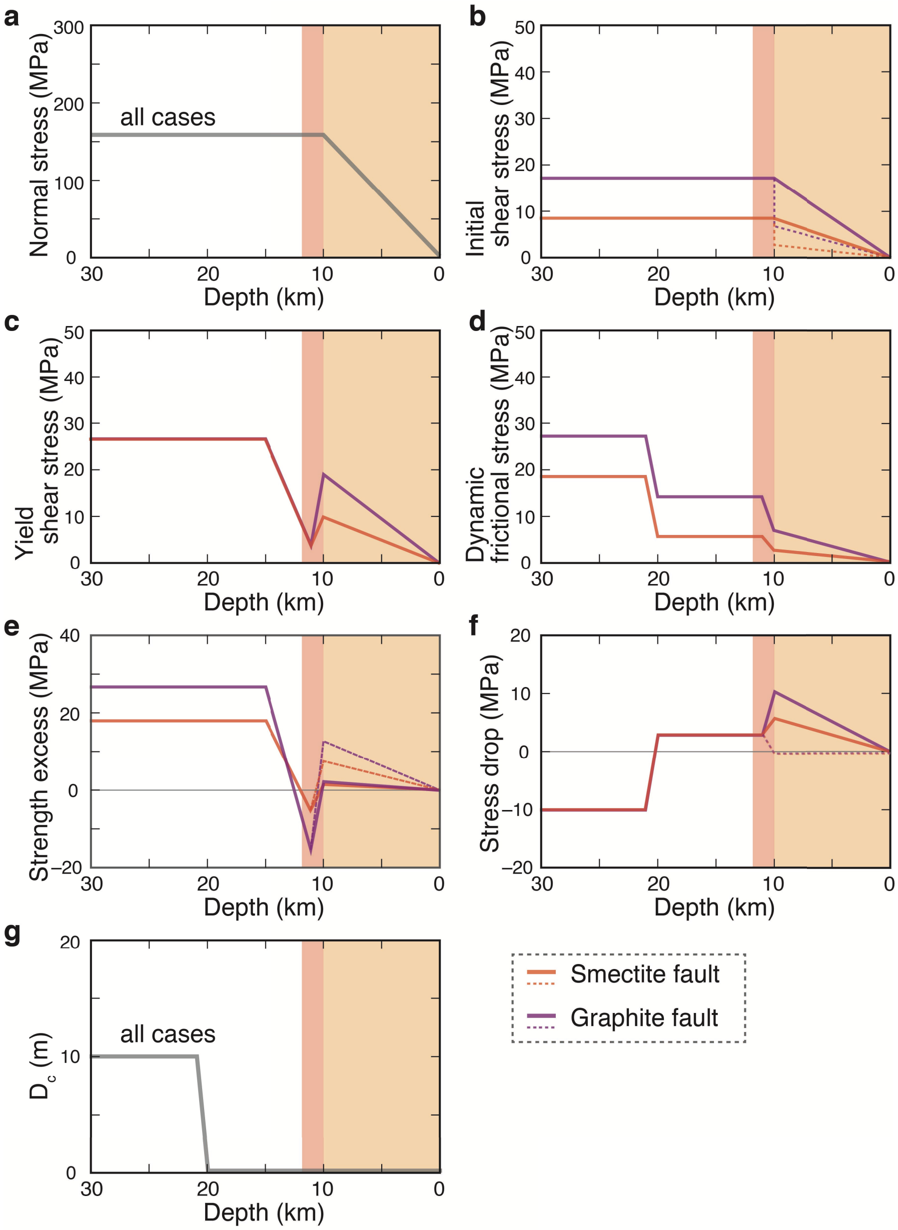

Supplementary Figure S2. Model parameters for dynamic rupture propagation for fault rocks composed entirely of either smectite or graphite. a, Normal stress. **b,** Initial shear stress. **c,** Yield shear stress. **d,** Dynamic frictional stress. **e,** Strength excess. **f,** Stress drop. **g,** D_c. Earthquake nucleation commenced at 11 km depth (pink shading) in response to a local reduction of yield stress. Model parameters in the shallow region (1–10 km depth, orange shading) are from our experimental data. The middle depth range of the fault (11–20 km) was assumed to have a stress drop of 3 MPa. In the region deeper than 21 km, a negative stress drop (–10 MPa) was assumed in order to prevent deeper rupture propagation. Dotted lines, models with negative stress drop (–0.2 MPa).

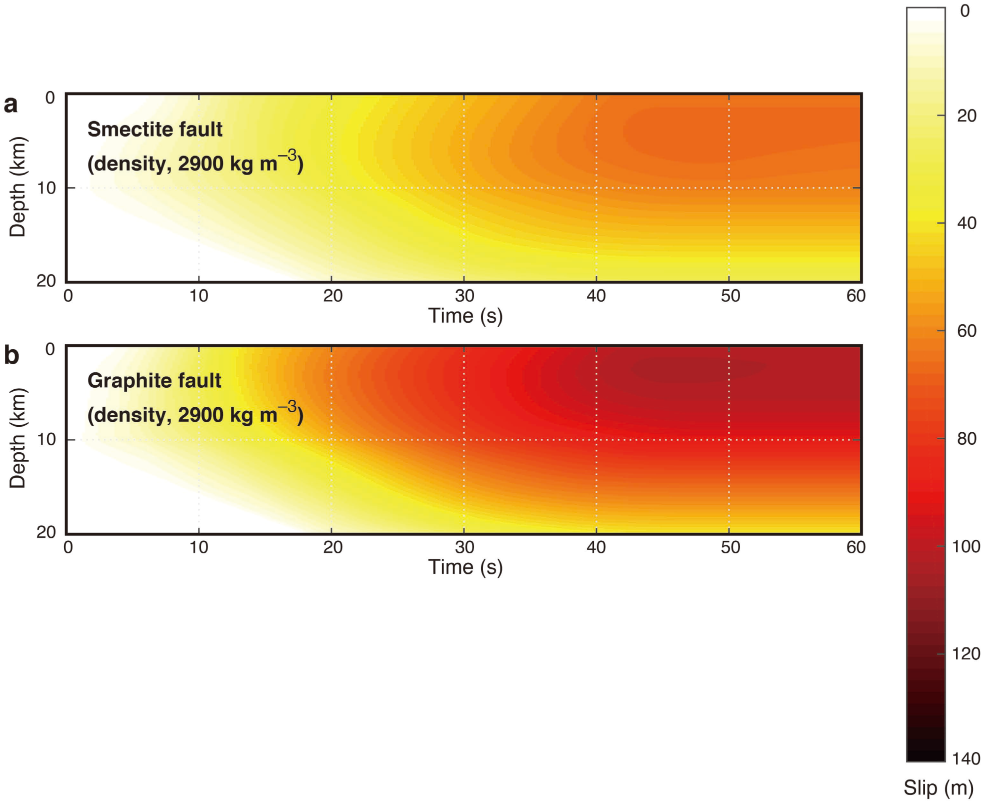

Supplementary Figure S3. Spatiotemporal evolution of slip for high absolute values of normal and shear stress. a, Smectite fault. **b**, Graphite fault.

By assuming a rock density of 2900 kg m^{-3}, effective normal stress calculated at 1 km depth was 18.7 MPa, 3 MPa higher than that for the original simulation (15.7 MPa) with rock density of 2600 kg m^{-3}. Initial shear stresses calculated at 1 km depth for the smectite and graphite faults were 1.03 and 2.06 MPa, respectively, higher than those of the original simulations (0.86 and 1.73, respectively) by 0.17 and 0.33 MPa, respectively. For both faults, these differences of stress values were added to the other stress parameters (initial shear stress, yield shear stress, and dynamic frictional stress) at all depths while keeping the same magnitudes of stress drop at all depths. The resultant slip distance for the smectite fault showed no difference from the original simulation (rock density 2600 kg m^{-3}), but the slip distance for the graphite fault was about 30 m shorter than that of the original simulation (rock density 2600 kg m^{-3}). The difference of the absolute stress level for the graphite fault (0.33 MPa) was greater than that for the smectite fault (0.17 MPa), indicating that higher absolute stress might result in higher shear resistance and shorter slip distance.

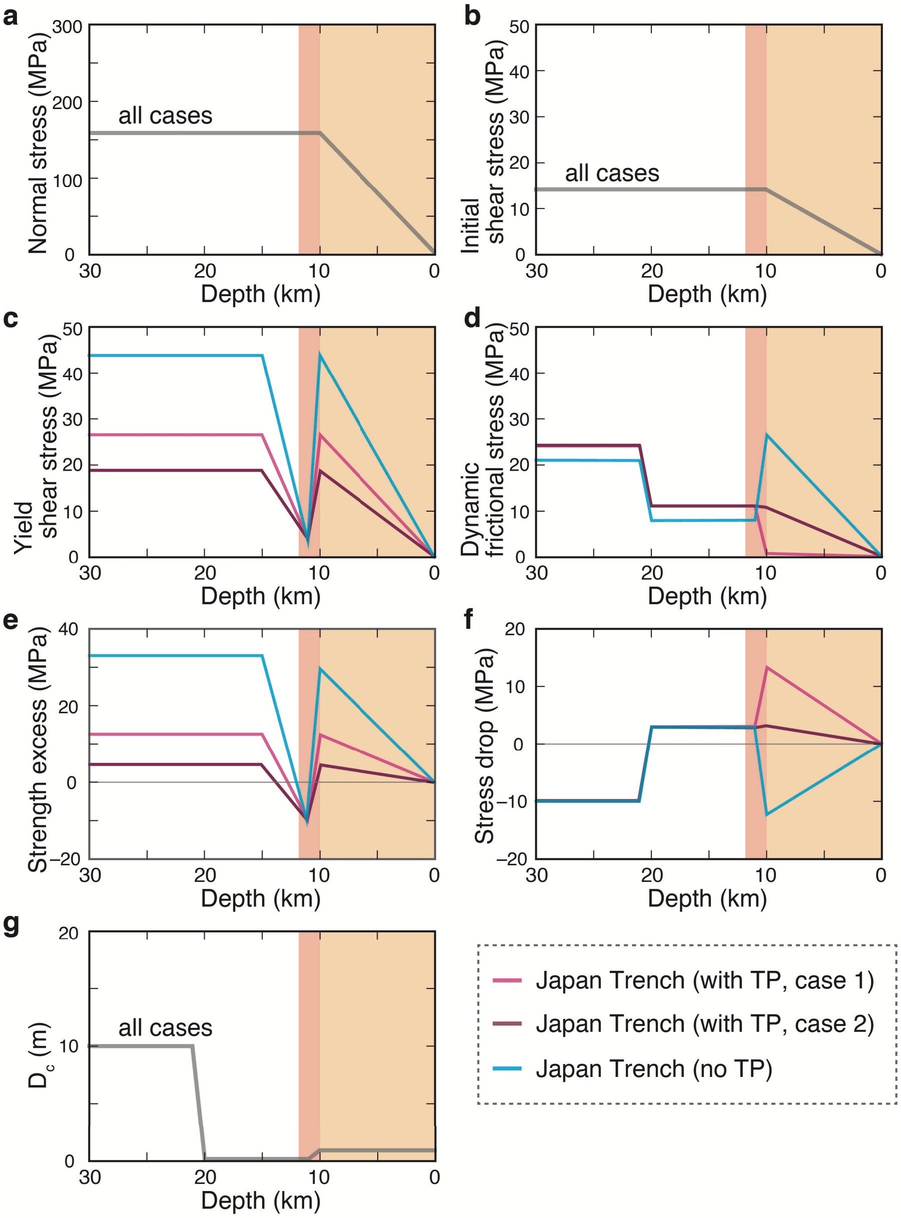

Supplementary Figure S4. Model parameters for dynamic rupture propagation for the Japan Trench. a, Normal stress. **b,** Initial shear stress. **c,** Yield shear stress. **d,** Dynamic frictional stress. **e,** Strength excess. **f,** Stress drop. **g,** D_c. Earthquake nucleation commenced at 11 km depth in response to a local reduction of yield stress (pink shading). Model parameters in the shallow region (1–10 km depth, orange shading) are from ref. 18 (with thermal pressurization, case 1), ref. 27 (with thermal pressurization, case 2), and ref. 11 (no thermal pressurization). The middle depth range of the fault (11–20 km) was assumed to have a stress drop of 3 MPa. In the region deeper than 21 km, a negative stress drop (–10 MPa) was assumed in order to prevent deeper rupture propagation. TP, thermal pressurization.

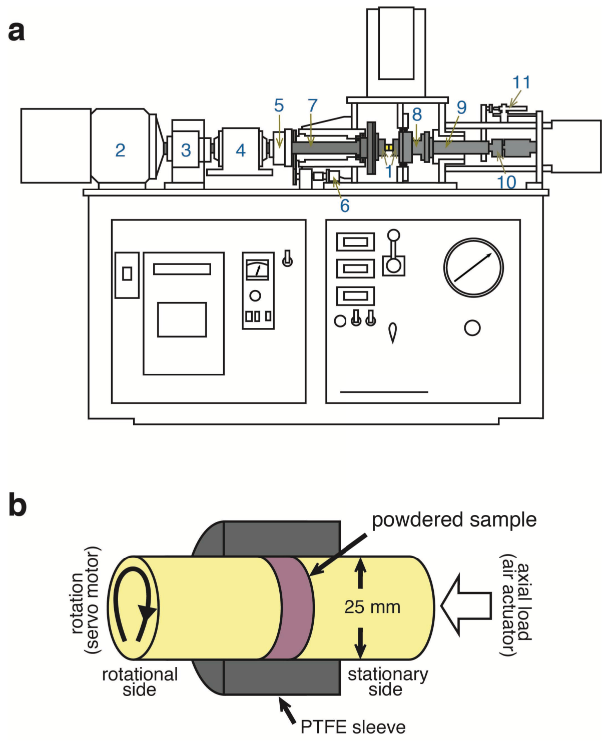

Supplementary Figure S5. Rotary shear apparatus used in this study. a, Sketch of the apparatus, modified from ref. 37. 1, specimen; 2, motor; 3, torque limiter; 4, torque gauge; 5, electromagnetic clutch; 6, rotary encoder; 7, rotary column; 8, torque-axial force gauge; 9, ball spline; 10, axial force gauge; 11, displacement transducer. **b,** Detailed sketch of sample assembly. PTFE, polytetrafluoroethylene. The two cylinders of Berea sandstone were 24.98 ± 0.01 mm in diameter and 20.00 ± 0.50 mm long. The inner surface of the PTFE sleeve was polished with #2000 emery paper and shaped to an inner diameter of 24.99 ± 0.02 mm.

Supplementary Reference:

37. Mizoguchi *et al.* High-velocity frictional behavior and microstructure evolution of fault gouge obtained from Nojima fault southwest Japan. *Tectonophysics* **471**, 285–296, (2009).
